# Disseminated Staphylococcus aureus Infection in an Adolescent Patient Following a Traditional Phlebotomy Procedure

**DOI:** 10.7759/cureus.44828

**Published:** 2023-09-07

**Authors:** Zhi Sing Oon, Ardilla Hanim Abdul Razak, Mohd Shukrimi Awang

**Affiliations:** 1 Department of Orthopedics, Traumatology and Rehabilitation, International Islamic University Malaysia, Kuantan, MYS

**Keywords:** infectious arthritis, wet cupping, phlebotomy therapy, acute osteomyelitis, bacterial abscess, methicillin-sensitive staphylococcus aureus

## Abstract

*Al-Fashdu is a* well-known Islamic medicine-based alternative treatment, and it has been widely practiced with the aim of treating certain health issues in various countries. Unfortunately, this therapy can lead to certain complications, including life-threatening infections. We report a case of a 12-year-old male patient who developed a disseminated Staphylococcus aureus infection following *Al-Fashdu* therapy. He was treated with surgical drainage and intravenous antibiotics. To our knowledge, this is the first report of a disseminated Staphylococcus aureus infection following *Al-Fashdu* therapy in an adolescent patient.

## Introduction

*Al-Fashdu* therapy is one of the alternative treatments well-known in the Islamic community. It is believed that this treatment was recommended by the Prophet Muhammad; in this therapy, blood is drained from the vein at chosen points [1]. There are two types of cupping methods: dry and wet cupping. While dry cupping is a non-invasive procedure with no bloodletting, wet cupping is an invasive method involving phlebotomy procedures [2]. In the literature, several cases of infections have been reported following cupping therapy. However, this is the first case report involving a disseminated methicillin-sensitive Staphylococcus aureus (MSSA) infection with concurrent septic arthritis of the ankle, osteomyelitis of the distal tibia, and MSSA bacteremia following wet cupping therapy in an adolescent patient; it was successfully treated with surgical drainage and intravenous antibiotics.

## Case presentation

A 12-year-old male presented to the emergency department (ED) with a complaint of painful right lower extremity swelling for three weeks. The history dated back to three weeks ago when he had suffered a right ankle sprain; he had visited the ED two days after the injury and had been discharged with analgesics. One week later, he sought treatment with alternative medicine due to unresolved ankle swelling and underwent a bloodletting and wet cupping procedure (Al-Fashdu). Subsequently, the swelling progressively worsened to the level of the proximal shin. The swelling was so painful that he had to resort to using a wheelchair to move around. The systemic review revealed that the patient had a history of chills and fever that had started with his aforementioned symptoms. The patient denied any other complaints suggestive of systemic infection, and his parents also denied any history of previous hospital admission, smoking, or drug abuse. Apart from his present complaint, he appeared to be a normal primary school student with normal development.

**Figure 1:**
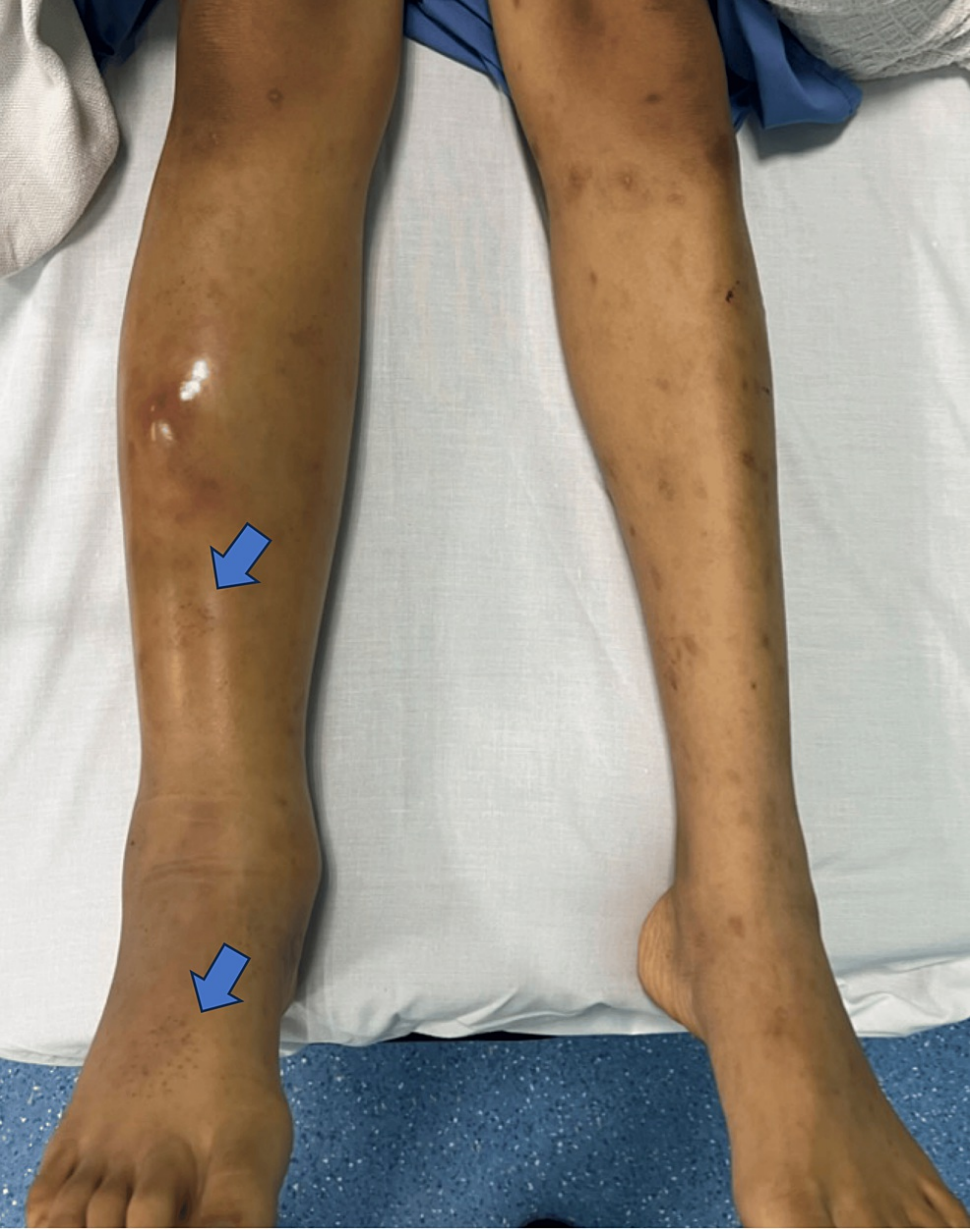
Clinical photo showing unilateral swelling of the right leg Multiple small punctured wounds (blue arrows) were seen on the anterior shin

Upon assessment at the ED, the patient had a Glasgow Coma Scale (GCS) score of 15; he clinically appeared ill, and his vital signs were as follows - temperature of 37.8 °C, blood pressure of 114/71 mmHg, tachycardic at 111 beats per minute, oxygen saturation of 100%, and respiratory rate of 22 breaths per minute. Local examination revealed a generalized swollen right lower limb extending from the foot until the knee level. There were multiple small punctured wounds on the anterior surface of the right shin and right foot (Figure 1). The lower limb was extremely tender to palpation, and the swelling was non-pitting in nature. There was an area of fluctuant swelling in the mid-shin, which was poorly demarcated. The range of motion test of the right ankle could not be performed due to severe pain. Otherwise, the right knee and hip range of motion were good. The neurovascular examination was normal.

Hematological examination showed an elevated white blood cell count (29.4 x 10^9^/L) with neutrophilia and mild anemia (92 g/L), and elevated thrombocytes (886 x 10^9^/L). There was a pronounced inflammatory response (erythrocyte sedimentation rate: 120 mm/hr; C-reactive protein: 15 mg/dL). Peripheral blood film confirmed iron deficiency anemia, leukocytosis, and reactive thrombocytosis. A peripheral blood culture was also taken. A summary of the laboratory investigations is shown in Table 1.

**Table 1:** Summary of the laboratory investigations

Parameters	Normal value	Result
Hemoglobin, g/L	115–155	92
Mean corpuscular volume, fL	77–95	76
Mean corpuscular Hemoglobin, pg	25–33	23.6
White blood cells, 10^9^/L	5.0–13.0	29.4
Neutrophils count, 10^9^/L	2.00–8.00	25.8
Platelet, 10^9^/L	180–400	886
Erythrocyte sedimentation rate, mm/hr	<21	120
C-reactive protein, mg/dL	<0.5	15.86
Sodium, mmol/L	136–146	125
Creatinine, umol/L	53–88	50

**Figure 2:**
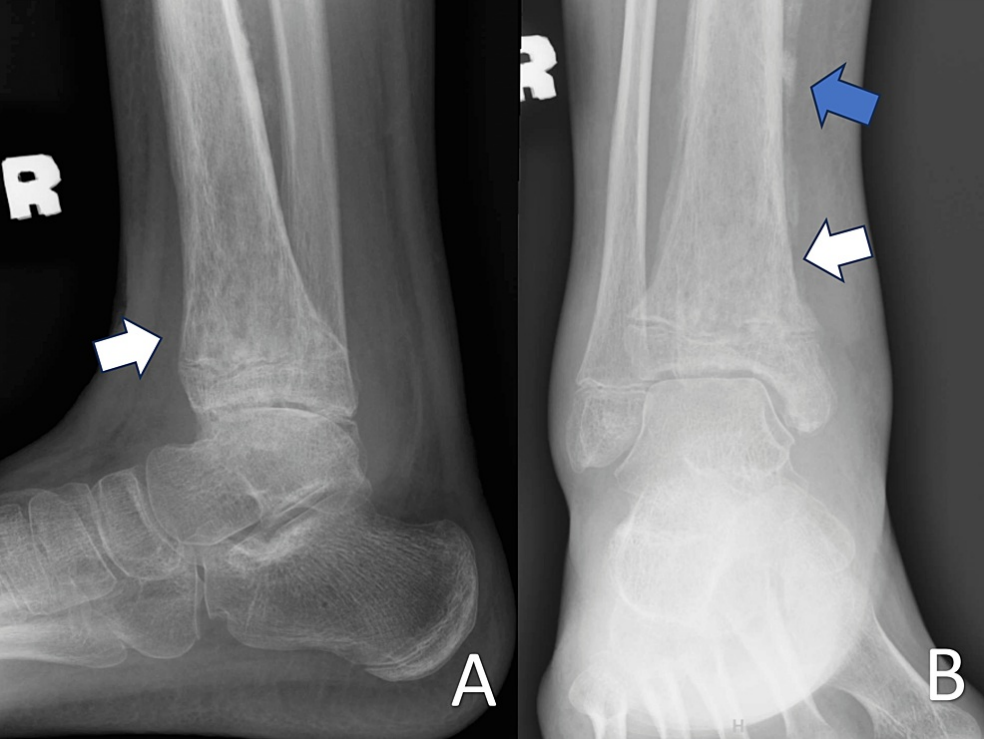
Plain radiograph of the left ankle in anteroposterior (A) and lateral (B) views The images revealed bony sclerosis (white arrows) and periosteal callus formation (blue arrow)

**Figure 3:**
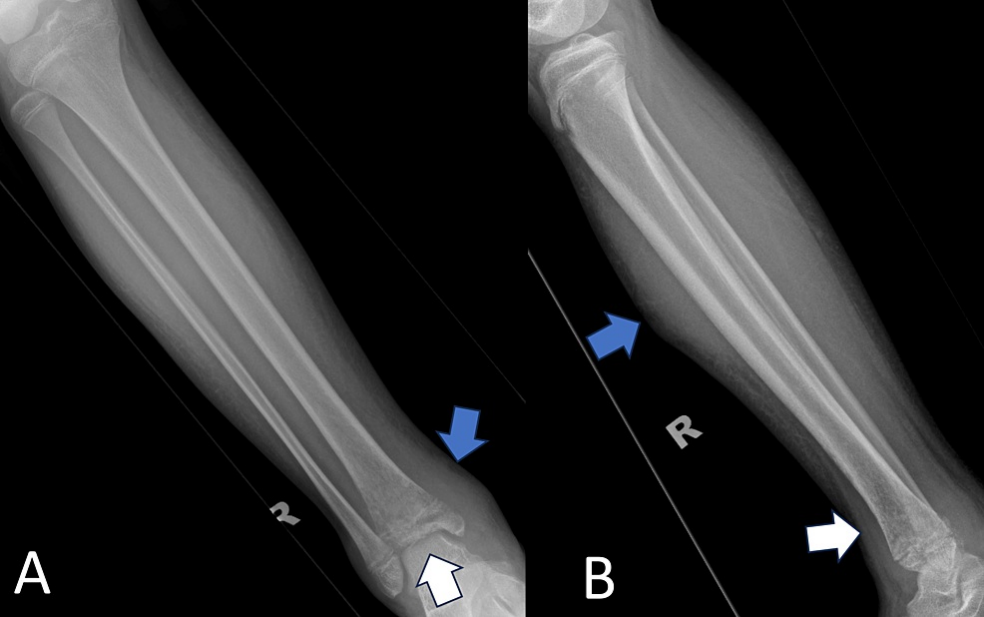
Plain radiograph of the right tibia in anteroposterior (A) and lateral (B) views The images showed an increase in soft tissue shadow and subcutaneous thickening (blue arrows) of the right leg. There was a loss of bony trabeculae and osteolysis changes at the distal metaphysis, physis, and epiphysis (white arrows)

**Figure 4:**
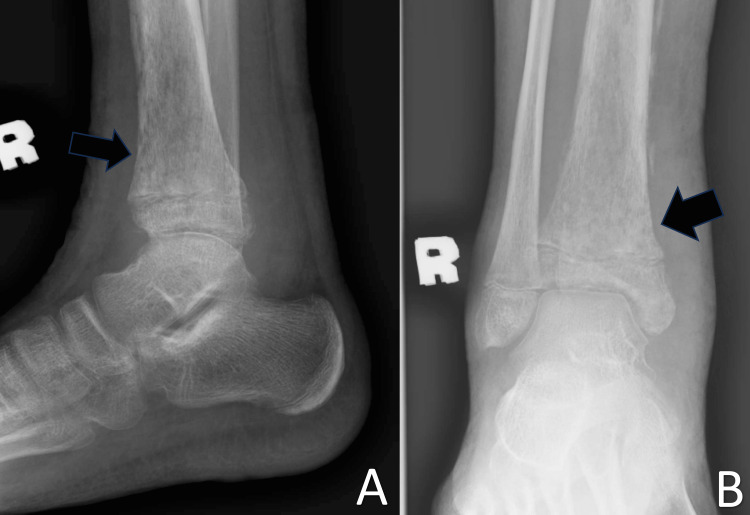
Plain radiograph of the right ankle in anteroposterior (A) and lateral (B) views The images revealed loss of bony trabeculae and osteolysis changes (black arrows)

**Figure 5:**
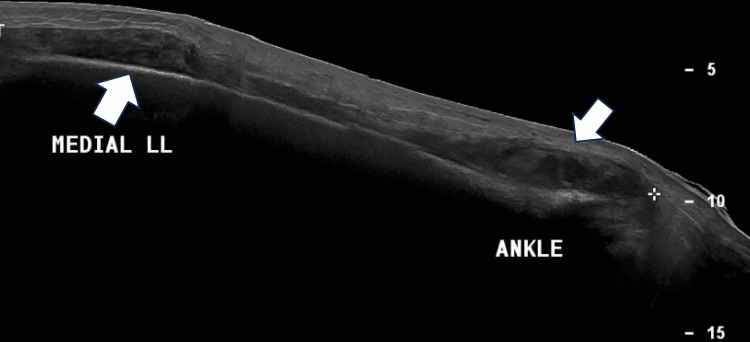
Ultrasound image of the right lower limb (LL) The image showed a deep-seated collection (white arrows) extending from the ankle to the proximal leg

A plain radiograph of the right tibia and right ankle revealed increased soft tissue swelling of the affected leg and loss of bony trabeculae with osteolysis changes seen at the distal metaphysis, physis, and epiphyseal region (Figures 2-4). A formal ultrasound reported a well-defined heterogenous intramuscular hypoechoic collection seen at the anteromedial aspect of the right shin, measuring approximately 1.8 x 4.5 x 26.5 cm, along with the presence of increased vascularity and mobile echogenic debris. The collection extended to the ankle region with intra-articular involvement of the right ankle joint (Figure 5).

Given the constellation of his clinical presentation and laboratory and radiological investigations, the patient was admitted with a diagnosis of right leg abscess with septic arthritis of the right ankle and osteomyelitis of the distal right tibia. He underwent incision and drainage of the leg abscess and arthrotomy washout of the right ankle. The patient was started on high-dose intravenous cloxacillin 2 grams every six hours to cover for MSSA, the most commonly encountered organism in osteoarticular infections among the pediatric population. The surgery was performed under general anesthesia. Intraoperatively, around 3 liters of hemopurulent discharge were removed. The infection could be tracked from the anterior compartment, breaching through the interosseous membrane, to the posterior compartment. The periosteal lining was intact, and the distal tibia was otherwise friable. The ankle was approached from the anterior aspect, and hemopurulent discharge was again drained upon breaching the capsule. Multiple tissue and purulent samples were taken for culture and sensitivity.

**Figure 6:**
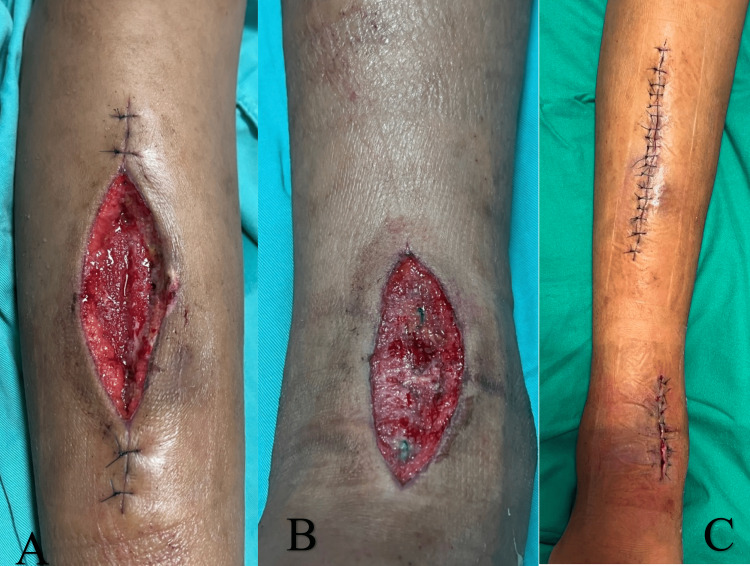
Clinical photos of the wound A and B: a clean wound base with granulation tissues. C: successful secondary suturing of the wound

Postoperatively, the patient was able to be extubated. His symptoms improved, with more than a 50% reduction in the pain score. The repeat blood investigations showed a significant reduction in the level of inflammatory markers (C-reactive protein: 3.0 mg/dL; white blood cell count: 12 x 10^9^/L). Multiple intraoperative tissues, intra-articular, bone samples, and peripheral blood cultures yielded MSSA organisms. The antibiotic was continued based on the result of drug sensitivity testing. Wound care was performed daily with an advanced dressing solution to keep it moist. Fortunately, after only one surgery, the limb edema had improved, and the wound condition was expectedly good; it showed healthy granulation tissue with no purulent discharge, and we were able to close all the wounds by secondary suturing (Figure 6).

**Figure 7:**
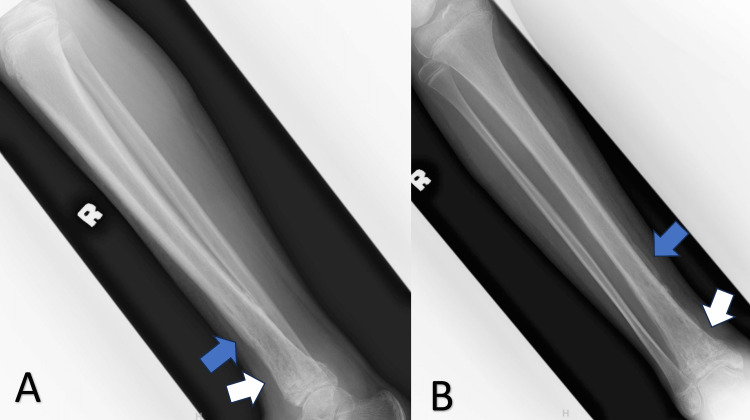
Plain radiograph of the right tibia in anteroposterior (A) and lateral (B) views The images revealed sclerosis (white arrow) and callus formation (blue arrow) from metaphysis to mid-shaft of the tibia

Fourteen days after the start of intravenous antibiotics, the patient's symptoms significantly improved. He was discharged from the hospital with a prescription for a minimum eight-week course of antibiotics. At the first follow-up after treatment, the wound was found to have healed and the inflammatory markers were close to normal values (C-reactive protein: <0.5 mg/dL; erythrocyte sedimentation rate: 69 mm/hr; white blood cell count: 7.7 x 10^9^/L). A repeat radiograph of the right tibia revealed sclerosis, periosteal callus formation, and the resolution of the osteolytic changes (Figure 7). Subsequent follow-ups were scheduled to monitor the secondary complications sustained from the infection.

## Discussion

The use of traditional and complementary medicine has expanded globally and has gained immense popularity. *Al-Fashdu* therapy has been described in the Hadiths of the Prophet Muhammad and it is mainly practiced among the Muslim community [1]. The principle behind *Al-Fashdu *is similar to wet cupping therapy, which involves phlebotomy on the superficial vein, followed by applying a heated cup to generate a partial vacuum that promotes blood flow and healing [3]. This therapy was believed by traditional medical practitioners to be able to remove metabolic waste products and toxins via deoxygenated blood from the veins [1].

Cupping therapy has been found beneficial in treating certain pain-related conditions, such as cervical spondylosis, acute gouty arthritis, and rheumatoid arthritis [4]. The safety of cupping therapy has been a matter of debate. Al Bedah et al. assessed the safety of cupping therapy and revealed that the most commonly reported adverse effects were scar formation and burning [5]. A systematic review of 135 cupping therapy-related randomized controlled trials by Cao et al. reported that the most common adverse effects included hematoma, pain, and paraesthesia at the treated site [6]. On the contrary, in our literature review, a total of 10 cases reported infections following cupping therapy [7]. These involved an even mix of both genders aged between 12 and 59 years. Eight patients had undergone wet cupping and two patients had dry cupping. Wang et al. have reported a case of a 12-year-old patient who suffered from an epidural abscess due to Stenotrophomonas maltophilia infection after a session of dry cupping at the lower back [8]. Multiple microorganisms were detected, including Staphylococcus aureus, Pseudomonas spp., Mycobacterium massiliense, and herpes simplex virus [7].

Pediatric musculoskeletal infections are common disorders and these can lead to significant disability. Concurrent osteomyelitis and septic arthritis are more common in newborns and adolescent age groups [9]. The ankle joint is the third most affected joint after the hip and knee [10]. The causative organisms can vary depending on the child’s age, immune status, and nutritional and vaccination status. Nevertheless, the most common organism causing acute hematogenous osteomyelitis and septic arthritis is Staphylococcus aureus, followed by Streptococcus species, Escherichia coli, and Pseudomonas aeruginosa [10,11]. These microorganisms can gain access to bone and joints via hematogenous spread, and direct inoculation from a contiguous focus of infection [12]. In our patients, Staphylococcus aureus organisms were yielded from blood cultures, intraoperative synovial tissues, and bone cultures.

Factors implicated as the sources of infection in traditional cupping therapy include personal hand hygiene of the traditional medicine practitioners, environmental hygiene of the workplace, sterility of the medical devices, and the patient's skin condition [13,14]. Koh et al. have reported an outbreak of skin and soft tissue infection in 109 patients following acupuncture procedures, owing to a contaminated batch of diluted glutaraldehyde solution used for disinfecting medical equipment [15]. In order to reduce the risk of procedure-related infections, a safety practice standard has been recommended [16]. In our patient, we believe that the traditional practitioner had not maintained a high standard of hygiene and sterility. The most likely route of the infection was a direct inoculation of Staphylococcus aureus organisms through the punctured skin during the wet cupping therapy, causing a local abscess formation, followed by hematogenous dissemination of infection into the adjacent ankle joint and distal tibia.

The treatment of osteoarticular infection involves the administration of antimicrobials and surgical drainage of abscesses. Traditionally, administration of at least two weeks of intravenous therapy was a common practice; however, a recent study has suggested shorter cycles with early transition to an oral antimicrobial regimen [17,18]. Discontinuing antimicrobial therapy is based on the normalization of inflammatory markers and clinical resolution of symptoms. The main sequelae of septic arthritis and osteomyelitis involving the physeal cartilage are joint stiffness due to secondary osteoarthritis, angular limb deformity, growth arrest, and limb length discrepancy [19]. Long-term regular follow-ups with repeat radiography are required for these patients [20].

## Conclusions

Cupping therapy is a widely used form of alternative medicine. Practitioners of cupping therapy should be aware of the potential complications that could occur following this procedure, particularly infections. Strict hygiene and sterilization practices should be maintained. Osteoarticular infection is an orthopedic emergency, and prompt surgical drainage and targeted antimicrobials are essential to reduce the incidence of long-term sequelae. A longer duration of follow-up is required in the current case for evaluating the possibility of growth disturbance.
